# Modeling the function of BAX and BAK in early human brain development using iPSC-derived systems

**DOI:** 10.1038/s41419-020-03002-x

**Published:** 2020-09-25

**Authors:** Piyush Joshi, Caroline Bodnya, Megan L. Rasmussen, Alejandra I. Romero-Morales, Anna Bright, Vivian Gama

**Affiliations:** 1 Vanderbilt Brain Institute, Vanderbilt University, Nashville, TN USA; 2 Department of Cell and Developmental Biology, Vanderbilt University, Nashville, TN USA; 3 Vanderbilt Center for Stem Cell Biology, Vanderbilt University, Nashville, TN USA

**Keywords:** Apoptosis, Cell death in the nervous system

## Abstract

Intrinsic apoptosis relies on the ability of the BCL-2 family to induce the formation of pores on the outer mitochondrial membrane. Previous studies have shown that both BAX and BAK are essential during murine embryogenesis, and reports in human cancer cell lines identified non-canonical roles for BAX and BAK in mitochondrial fission during apoptosis. BAX and BAK function in human brain development remains elusive due to the lack of appropriate model systems. Here, we generated BAX/BAK double knockout human-induced pluripotent stem cells (hiPSCs), hiPSC-derived neural progenitor cells (hNPCs), neural rosettes, and cerebral organoids to uncover the effects of BAX and BAK deletion in an in vitro model of early human brain development. We found that BAX and BAK-deficient cells have abnormal mitochondrial morphology and give rise to aberrant cortical structures. We suggest crucial functions for BAX and BAK during human development, including maintenance of homeostatic mitochondrial morphology, which is crucial for proper development of progenitors and neurons of the cortex. Human pluripotent stem cell-derived systems can be useful platforms to reveal novel functions of the apoptotic machinery in neural development.

## Introduction

The intrinsic cell death pathway can be initiated by various stimuli including metabolic stress and exposure to cytotoxic agents. The response to these stimuli is mediated by the B-cell lymphoma 2 (BCL-2) family, including proapoptotic and antiapoptotic members that are evolutionarily conserved^1^. During steady state, antiapoptotic members, which include BCL-2, B-cell lymphoma-extra-large (BCL-XL), and myeloid cell leukemia 1 (MCL-1) preserve the integrity of the outer mitochondrial membrane by keeping the proapoptotic effectors Bcl-2-associated X protein (BAX) and Bcl-2 homologous antagonist/killer (BAK) in an inactive state^2,3^. Once activated, BAX and BAK form pores within the mitochondrial outer membrane causing mitochondrial outer membrane permeabilization and release of cytochrome c^4–9^. Cytochrome c then binds to apoptotic peptidase, activating factor 1, and caspase-9 to form the apoptosome initiating a caspase cascade that ultimately leads to cell death^8^.

Mouse models lacking BAX or BAK present with mild defects in development. BAX-deficient male mice are sterile due to an arrest in spermatogenesis resulting from ineffective developmental apoptosis. Despite this, animals lacking BAX are viable^9^. BAK, which is closely related to BAX in assayed in vitro systems^10–12^, displays widespread tissue distribution similar to BAX. BAK-deficient mice also show normal development, suggesting BAK has redundant functions with other proapoptotic BCL-2 family members^13^. Only 10% of mice lacking both BAX and BAK survive to adulthood. The surviving mice show multiple phenotypic abnormalities ranging from interdigital webs to imperforate vaginas to neurological abnormalities^13^. Mice lacking BAX, BAK, and Bcl-2 related ovarian killer (BOK), which has been recently implicated as an effector with genetic, biochemical, and structural studies^6,14–20^, are unable to undergo intrinsic apoptosis. These BAX/BAK/BOK triple knockout (TKO) mice show severe defects compared to BAX/BAK double knockout (DKO) mice and only 1% of mice survive to adulthood^16^. These previous studies suggest BAX, BAK, and BOK represent redundant proteins involved in regulation of apoptosis; however, their roles have not been well studied in human model systems. Human induced pluripotent stem cell (hiPSC) model systems represent new tools that can provide insight into the function of the BCL-2 family in human development.

In addition to the canonical roles of BAX and BAK in apoptosis, recent studies^21–26^ have demonstrated non-canonical functions for these proteins in regulation of mitochondrial dynamics and morphology^21–23,25,27,28^. Mitochondria are highly dynamic organelles that continuously cycle through fission and fusion to modulate mitochondrial morphology. Dysregulation of these fundamental processes have been implicated in diseases ranging from diabetes to neurodegeneration^29^. The balance of fission and fusion is regulated by several GTPases that maintain mitochondrial length and connectivity. Mitochondrial fusion is primarily coordinated by GTPases Mitofusin 1, Mitofusin 2 (MFN2), and Optic atrophy protein 1 (OPA1), which fuse the outer and inner mitochondrial membranes^30–33^. Fission is mediated mainly by Dynamin-related protein 1 (DRP1) which divides the outer and inner membranes of the mitochondria^34–36^. It has been proposed that BCL-2 proapoptotic proteins contribute to mitochondrial morphogenesis in healthy cells^37^. The soluble form of BAX stimulates fusion in a MFN2-dependent manner^25^, while BAX/BAK-deficient cells have been described in some reports to have constitutive defects in mitochondrial morphology^23^. BAX has been associated with mitochondrial fission by colocalizing with DRP1 during apoptosis^22^, but there are limited studies assessing the function of BAX in mitochondrial dynamics during homeostatic conditions in the context of human brain development.

Previous studies with hiPSCs and differentiated cells demonstrated the significant remodeling of the mitochondrial network as cells undergo differentiation or reprogramming^38,39^. The mitochondrial priming state—how close a cell is to the threshold of apoptosis—is also reported to reset during differentiation^40,41^. BAX is constitutively active at the Golgi in human embryonic stem cells^42^, while in differentiated cells, inactive BAX localizes to the cytosol. These dramatic changes in mitochondrial morphology, dynamics, and apoptotic sensitivity, as well as their ability to differentiate, make hiPSCs an attractive model for studying the effects of BAX and BAK deletion on mitochondrial morphology and developmental apoptosis.

In this study, hiPSCs and hiPSC-derived neural progenitor cells (hNPCs), neural rosettes, and cerebral organoids were used to model in vitro the effects of BAX and BAK deficiency in early human brain development. Previous studies have not assessed the effects of BAX and BAK in hiPSC model systems nor their effects on early brain development or mitochondrial function. BAX and BAK DKO hiPSCs were generated using clustered regularly interspaced short palindromic repeats-CRISPR-associated protein 9 (CRISPR-Cas9) mediated gene editing. We examine whether BAX and BAK regulate mitochondrial morphology during homeostatic conditions without an apoptotic stimulus. Structured illumination microscopy (SIM) of the mitochondrial network of BAX/BAK DKO hiPSCs and hNPCs revealed severe loss of mitochondrial structure. In addition, human neural rosettes and cerebral organoids that serve as three dimensional (3D) models of early human brain development showed that BAX and BAK deficiency leads to abnormal corticogenesis. Taken together, these data indicate that BAX and BAK may be key modulators of mitochondria morphology and early brain development in human model systems.

## Methods

### hiPSCs cell culture

GM25256 (Allen Institute, Seattle, WA) hiPSCs are from a 30-year-old healthy male and cultured in mTeSR1 or E8 stem cell maintenance media. hiPSCs were dissociated by incubation for 4 min in Gentle Cell Dissociation Reagent (StemCell Technologies, Vancouver, BC) for splitting and replating. Cells were plated on matrigel-coated (BD Biosciences, San Jose, CA) six-well plates for tissue culture, Seahorse XF96 cell culture microplates for mitochondrial function assay, 35 mm immunofluorescence glass bottom plates for imaging, and 96-well plates for CellTiter-Blue (CTB) and cleaved caspase assays.

### hNPCs differentiation

hiPSCs were dissociated using Gentle Cell Dissociation Reagent and plated at 2.5 × 10^6^ cells/ml with STEMdiff Neural Induction Medium (Stem Cell Technologies) and 10 µM ROCK inhibitor Y-27632 (Tocris) on Matrigel-coated six-well plates for differentiation. After passage one, hNPCs were replated on seahorse XF96 cell culture microplates for mitochondrial function assay, 35 mm immunofluorescence glass bottom plates for imaging, and 96-well plates for CTB and cleaved caspase assays.

### Neuralized embryoid body (EB) and neural rosette differentiation

hiPSCs were plated at a concentration of 3.0 × 10^6^ cells/well with E6 media into Aggrewell plates pretreated with 500 µl of Aggrewell Rinsing Solution (StemCell Technologies, Vancouver, BC). On day 4, embryoid bodies were replated into 35 mm Matrigel-coated immunofluorescence plates, and neural rosettes were fixed for imaging on day 8.

### Cerebral organoid differentiation

hiPSCs were plated at 2.7 × 10^6^ cells/well with embryoid body formation medium (STEMdiff Cerebral Organoid Basal Media 1 and STEMdiff Cerebral Organoid Supplement A; StemCell Technologies, Vancouver, BC) into Aggrewell plates pretreated with 500 µl of Aggrewell Rinsing Solution. Between days 0 and 3, cells were maintained in embryoid body formation medium and media was changed on day 2. On day 4, EBs were replated into 10 cm tissue culture dishes pretreated with Aggrewell Rinsing Solution. Between days 5 and 7, EBs were maintained in induction medium: STEMdiff Cerebral Organoid Basal Media 1 and STEMdiff Cerebral Organoid Supplement B. On day 7, the EBs were embedded in Matrigel with expansion medium: STEMdiff Cerebral Organoid Basal Media 2, STEMdiff Cerebral Organoid Supplement C, and STEMdiff Cerebral Organoid Supplement D. Between days 7 and 10, EBs were maintained in expansion medium. On day 10, the Matrigel coat was broken and the organoids were moved back into 60 mm ultralow attachment tissue culture dishes. Between days 10 and 60, organoids were maintained in maturation medium: STEMdiff Cerebral Organoid Basal Media 2, STEMdiff Cerebral Organoid Supplement E, and 100X Penicillin-Streptomycin. More information about this protocol has been previously described^43^.

### CRISPR-mediated gene editing

On the day of the transfection, the cells were counted to 1 × 10^6^ cells per reaction. 2 µl Cas9 Nuclease (1 µg/µl) and 6 µl of gRNA designed to target BAX and BAK with a sequence complementary to the target DNA were added to 1.5 ml tubes. Cells were mixed in Resuspension Buffer R and the mixture was incubated at room temperature for 5–10 min (this is the Cas9/gRNA complex solution). The Neon Transfection System was then set up by filling the Neon Tube with 3 ml of Electrolytic Buffer (Buffer E2 for 100 µl Neon Tip) and the Neon Tube inserted into the Neon Pipette station. The 100 µl of Cas9/gRNA cell mix was aspirated with the 100 µl Neon Tip. The cells were then electroporated and immediately transferred to a 6-well plate with 2 ml of pre-warmed StemFlex media (Thermo Fisher, Waltham, MA). Cells were incubated at 37 °C for 48–72 h. Cell population was then DNA sequenced to determine indels.Cells were flow-sorted by single cell into a 96-well plate. They were then cultured for two weeks; their DNA was collected and sequenced. Immunoblots were also conducted to confirm that the protein had been knocked out.

### Carbonyl cyanide m-chlorophenyl hydrazone (CCCP) and etoposide exposures

hiPSCs and hNPCs were exposed to the mitochondrial and DNA-damaging agents CCCP and etoposide. The results were evaluated with the cell viability CellTiter-Blue and Caspase-Glo 3–7 cleaved caspase-3 activation assays (Promega, Madison, WI). hiPSCs were replated at 100,000 cells/ml for caspase assay and 500,000 cells/ml for cell viability assay. The day after replating, media was changed, and the 24-h CCCP or etoposide exposures were started 2 days after replating. All cells were plated into Matrigel-coated 96-well plates and exposed to CCCP/etoposide in cell type appropriate medium.

### Caspase-Glo 3/7 assay

After the 24-h etoposide/CCCP exposure, 25 µl of Caspase-Glo 3/7 Reagent (Promega, G8090) and 25 µl of cell type appropriate medium was added to each well of the 96-well plate. In this assay, reagent addition results in cell lysis, caspase cleavage of the substrate, and a luminescent signal produced by luciferase. Luminescence is proportional to caspase-3 and -7 activity levels. The luminescence was measured with a luminometer 1 h after reagent addition. The sample size (*n*) refers to the number of independent replatings and exposures within each group.

### CellTiter-Blue (CTB) cell viability assay

After the 24-h etoposide/CCCP exposure, 20 µl of CTB reagent (Promega, G8081) was added to each well of the 96-well plate. Cell lysis buffer (10% Triton in phosphate-buffered saline [PBS]) was added to some wells to provide background fluorescence for 0% viability. The fluorescence was measured with an excitation of 570 nm and emission of 600 nm using a Beckman Coulter DTX 880 multimode plate reader 2 h after reagent addition (Beckman Coulter, Brea, California). The sample size (*n*) refers to the number of independent replatings and consequent exposures within each group.

### Immunoblot

Protein samples were collected in lysis buffer: 1% Triton, 100x phenylmethylsulfonyl fluoride, 10X phosphatase inhibitor (PhosSTOP), and 10X protease inhibitor cocktail (Sigma Technologies, Perrysburg, OH), and quantified using BCA analysis (Thermo Fisher, Pierce, Pittsburgh, PA). The samples (50 µg each) were boiled at 95 °C and loaded into a 12% gel, which was then transferred to a nitrocellulose membrane and stained with primary (1:1000 BAX, BAK; Cell Signaling Technologies, Danvers, MA) and Li-Cor Odyssey secondary antibodies (1:10,000). β-actin (Sigma Technologies, Perrysburg, OH) was used as a control to ensure differential loading across lanes was not a confounding variable. Membranes were imaged using the Li-Cor Odyssey Imaging System, and quantification was performed using Image Studio Lite (Li-Cor, Lincoln, NE).

### Immunofluorescence and image acquisition

To prepare for immunofluorescence, cells were fixed with 4% PFA in 1X PBS at room temperature for 20 min. Cells were then washed with PBS, permeabilized with 1% Triton X-100 in PBS for 5 min at room temperature and washed with 1X PBS again. Cells were then blocked with 10% BSA in PBS for 20 min at room temperature, and primary antibody in 10% BSA was added and incubated overnight at 4 °C. Anti-TOM20 antibody was used for staining of the outer mitochondrial membrane (Cell Signaling Technologies, 1:200), and anti-Mitochondria (Anti-Mito) antibody was used for visualization of mitochondrial morphology (Abcam, 1:200). Primary antibodies were then washed out three times with 1X PBS. Secondary antibodies were added to the cells in 10% BSA for 1 h at room temperature and the cells were then washed three times with 1X PBS. Hoechst 33528 (10 mg/ml stock at 1:10,000 dilution in PBS) was added for 5 min at room temperature, and the cells were washed with 1X PBS again. The cells were then mounted in Fluoromount-G mounting medium (Thermo Fisher Scientific). Images were acquired using a Nikon SIM equipped with a 1.49 NA ×100 Oil objective and Andor DU-897 EMCCD camera. Quantification of mitochondrial morphology was performed in NIS-Elements (Nikon); briefly, we segmented mitochondria in 3D and obtained volume and object count data from the resulting 3D mask. Measurements were exported into Excel and data was analysed using Prism 8.

Immunofluorescence was also conducted in neural rosettes derived from hiPSCs. To prepare for immunofluorescence, cells were washed twice with PBS, then fixed with methanol at −20 °C for 10 min. They were then washed with PBS and blocked at room temperature with 5% Donkey Serum (Fisher 5058837) + 0.3% Triton X-100 in tris buffered saline (TBS). Primary antibody was then added (ZO-1 1:500, CDK5RAP2 1:200, Tubulin 1:500) in 5% Donkey Serum + 0.3% Triton X-100 in TBS overnight. After washing three times with TBS, secondary antibody was added (1:500) in 5% Donkey Serum + 0.3% Triton X-100 in TBS for 2 h, which was then washed off three times with TBS. Hoechst 33528 (1:10,000) in TBS was then added and the cells incubated at room temperature in the dark for 5 min. After three more TBS washes, cells were mounted in Fluoromount-G mounting medium. Images were acquired with an Andor DU-897 EMCCD camera mounted on a Nikon Spinning Disk Microscope equipped with 0.45 NA ×10 and 0.75 NA ×20 objectives. Data analysis was performed using NIS-Elements (Nikon). A Neural Rosette Quantification macro was utilized to measure the rosette lumen area based on the ZO-1 staining channel. Measurements were exported into Excel and data was analysed using Prism 8.

Immunofluorescence for cerebral organoids was conducted after cryo-sectioning of samples. The cells were then blocked in PBS + 5% donkey serum + 0.2% Triton X-100 and incubated at room temperature for 1 h. Primary antibodies SOX2 (Cell Signaling Technologies, 5049S), TBR1 (Abcam, AB31940), CTIP2 (Abcam, AB18465), PAX6 (Cell Signaling, D3A9V), REELIN (Millipore, MAB5366), TBR2 (Millipore, AB15894), βIII-Tubulin (TU-20) (Cell Signaling, 4466S), and TOM20 (Cell Signaling, D8T4N) diluted in blocking solution (1:1000) were then added overnight at 4 °C. The cells were then washed three times with PBS + 0.1% Tween-20 (PBST) and respective secondary antibodies diluted in PBST with 5% donkey serum were added at RT in the dark for 1 h. The cells were washed again 3X with PBST and counterstained with Hoechst 33528 (1:10,000) in PBS. After three more PBS washes, mounting media was added. Confocal images of the organoids were acquired using an Andor DU-897 EMCCD camera mounted on a Nikon Spinning Disk Microscope with 0.45 NA ×10 and 0.75 NA ×20 air objectives (macro structures) and a 1.49 NA ×100 Oil objective (mitochondria imaging). The software used for image acquisition and reconstruction was NIS-Elements (Nikon) and Image processing was performed using Fiji/ImageJ^44^.

### Apoptosis/necrosis detection assay

An apoptosis/necrosis detection assay (Abcam, Cambridge, UK) was conducted to determine levels of apoptotic or necrotic activity in hiPSCs and hNPCs. Cells were first plated at 100,000 cells/ml in 35 mm immunofluorescence plates. When they reached 70–80% confluency, the assay was conducted. The cells were washed two times with 100 µl of Assay Buffer and then resuspended in 200 µl of Assay Buffer. In total, 2 µl of 100X Apopxin Green Indicator for detecting apoptotic cells, 1 µl of 200× 7-aminoactinomycin D (7-AAD) for detecting necrotic cells, and 1 µl of 200x CytoCalcein Violet 450 for detecting healthy cells were added. The cells were then incubated for 30–60 min and the cells were washed two times with 150 µl Assay Buffer and resuspended in 200 µl Assay Buffer before imaging using a Nikon Eclipse Ti inverted widefield microscope and quantification of apoptosis/necrosis levels.

### Seahorse assay

The Seahorse Cell Mito Stress Test (Agilent, Santa Clara, CA) was conducted to assess mitochondrial function. Two days prior to the assay, hiPSCs were replated in E8 media and hNPCs were replated in E6 media at 500,000 cells/ml on Seahorse XF96 cell culture microplates (Agilent). One day before the assay, Seahorse XFe96 extracellular flux assay cartridge (Agilent) was hydrated with 200 μl/well of water in a non-CO2 incubator overnight. On the day of the assay, Seahorse XF Calibrant was added to Seahorse XFe96 extracellular flux assay cartridge for one hour before loading drugs. Seahorse medium (Agilent) with 1 mM pyruvate, 2 mM glutamine, and 10 mM glucose warmed to 37° was added to the cells and the plate was incubated in a non-CO2 incubator for 1 h before the assay. Appropriate concentrations of oligomycin (1.5 µM), FCCP (1.5 µM), and Rot/AA (0.5 µM) were added to Seahorse XFe96 extracellular flux assay cartridge and cartridge was loaded into XF Extracellular Flux Analyzer. After calibration step, cell plate was loaded into the XF Extracellular Flux Analyzer to assess mitochondrial function.

### Statistical analyses

Statistical analyses were performed using Prism software version 8.0 (GraphPad, La Jolla, CA) and Excel (Microsoft, Redmond, WA). Two-way ANOVA with Tukey’s multiple comparisons test was conducted for apoptosis/necrosis assay. Two-way ANOVA was performed using Prism software, and Tukey’s comparisons tests were used for binary post hoc comparisons for 24-h etoposide and CCCP exposures in hiPSCs and hNPCs. Wilcoxon test was conducted for hiPSCs and hNPCs mitochondrial volume. Ordinary one-way ANOVA with Tukey’s post hoc was used for EB size and neural rosette lumen size.

## Results

### Validation of BAX/BAK DKO hiPSCs cell death phenotypes in response to DNA and mitochondrial toxicants

**Fig. 1:**
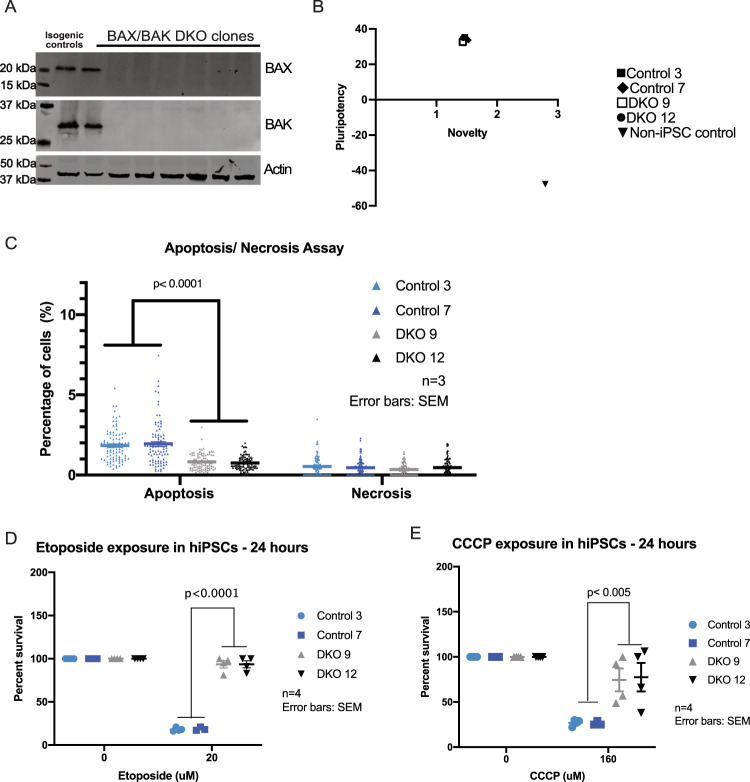
Characterization and validation of BAX/BAK DKO hiPSCs cell death phenotypes. **a** Immunoblot shows complete KO of BAX and BAK in DKO clones and full expression of proteins control clones normalized to beta-actin. **b** Pluritest assay shows two control and two DKO clones as being pluripotent compared to non-iPSC control. **c** Apoptosis/necrosis assay shows that DKOs have significant decrease in apoptosis after three independent experiments. Error bars: SEM. Each symbol represents percentage of cells in the field of view that were positive for apoptosis or necrosis. **d** hiPSCs DKO clones demonstrate increased cell survival with 24-h 20 µM DNA-damaging etoposide compared to control clones as shown by two-way ANNOVA. **e** hiPSCs DKO clones have increased cell survival compared to control when exposed for 24 h with 160 µM CCCP. Four independent experiments were conducted for etoposide and CCCP exposures. Each symbol represents an individual experiment and the percent survival for that clone. Error bars: SEM.

We first sought to generate BAX and BAK DKO and isogenic controls using CRISPR-Cas9-mediated gene editing as previously described^45^ (Supplementary Fig. 1A). The deletion of BAX/BAK in hiPSCs was validated by immunoblot (Fig. 1a) and DNA sequencing. We determined that BAX/BAK DKO hiPSCs did not have karyotype abnormalities (Supplementary Fig. 1B) and remained pluripotent after gene deletion using a Pluritest Assay (Fig. 1b). The Pluritest Assay compares the transcriptional profile of samples to an extensive reference set of over 450 cell and tissue types. The pluripotency score indicates how strongly the sample matches model bases pluripotency signature, and the novelty score indicates general model fit of sample compared to reference set of samples.

As expected, a significant decrease in basal apoptosis was detected in DKO iPSC clones compared to controls when stained for Apopxin Green Indicator (Fig. 1c). To confirm that BAX and BAK deletion did not result in the induction of basal necrosis, hiPSCs were stained for 7-AAD. No difference was observed in levels of necrosis after quantification (Fig. 1c). Next, isogenic controls and DKO clones were exposed to 20 µM etoposide, an inhibitor of topoisomerase II which causes double stranded breaks. After 24 h of treatment, a CTB assay was conducted to validate the decrease of cell death in DKO iPSC cells (Supplementary Fig. 2A). DKO iPSC clones are significantly more resistant to the DNA-damaging agent than control cells (Fig. 1d). Isogenic controls and DKO iPSC clones were also exposed to 160 µM CCCP, a mitochondrial toxicant that inhibits oxidative phosphorylation by uncoupling the proton gradient. After the 24-h exposure, CTB assay was conducted and data showed that DKO clones were also more resistant to mitochondrial toxicity (Fig. 1e), as widely established in previous studies^46–49^. Cleaved caspase-3 assays were also conducted in hiPSCs after exposure to etoposide and CCCP (Supplementary Fig. 2B, C). They show the same effect as observed in CTB assays.

### BAX/BAK DKO hNPCs maintained the cell death resistance phenotype

**Fig. 2:**
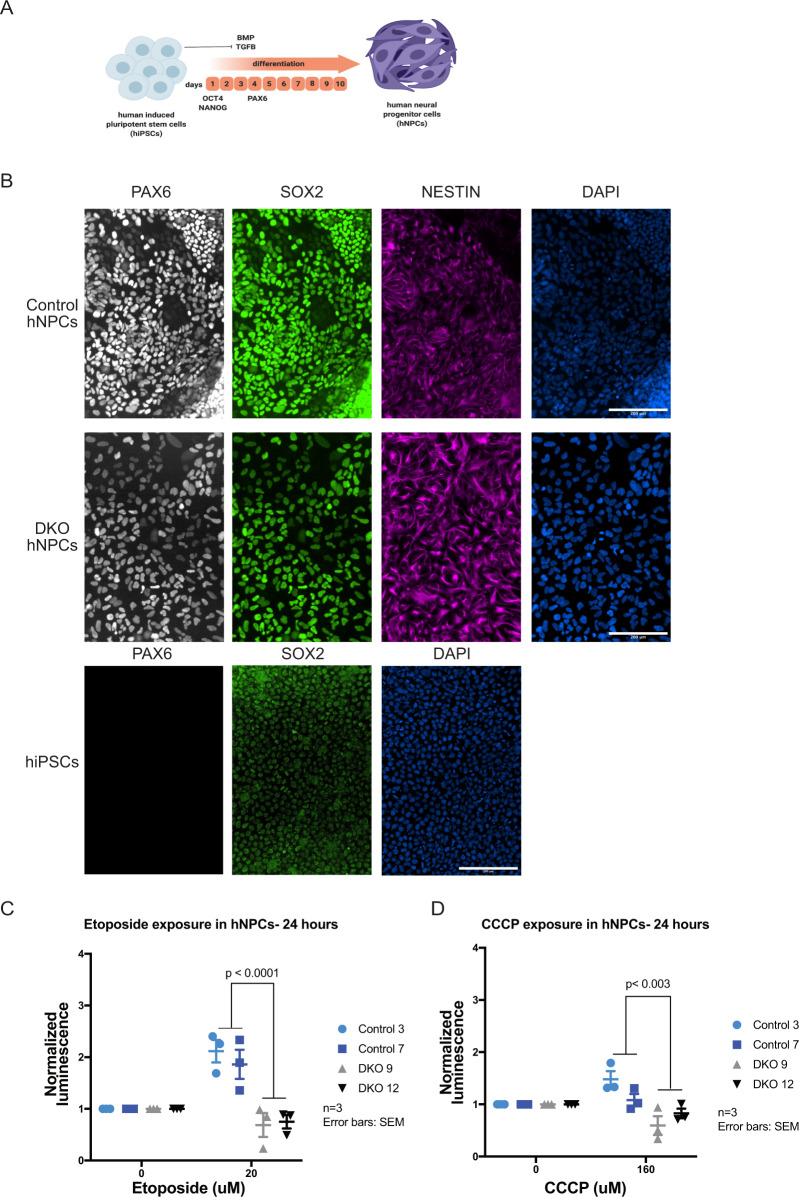
Differentiation of BAX/BAK DKO into hNPCs and validation of cell death phenotype. **a** Diagram of hiPSCs to hNPCs differentiation protocol based on inhibiting BMP and TGFB pathways. **b** Derived hNPCs were stained for neural progenitor markers PAX6, SOX2, and Nestin to validate. hiPSCs were also stained for PAX6 and SOX2 to validate. **c** hNPCs DKO clones have decreased cleaved caspase-3 activity due to loss of BAX and BAK with 24-h etoposide exposure. **d** hNPCs DKO clones demonstrate a decrease in CC3 compared to controls with 24-h CCCP exposure. Three independent experiments were conducted for etoposide and CCCP exposures. Error bars: SEM.

In order to validate the cell death phenotypes in differentiated neuronal cells, isogenic controls and DKO iPSCs were differentiated into hNPCs using dual SMAD inhibition with STEMdiff Neural Induction Medium (Stem Cell Technologies) (Fig. 2a). The differentiation to hNPCs was validated using immunofluorescence for the neural progenitor markers PAX, SOX2, and Nestin (Fig. 2b). Quantification of PAX6 and SOX2 shows similar levels across control and DKO hNPCs (Supplementary Fig. 3A). As with hiPSCs, hNPCs were exposed to high doses of etoposide (20 µM) and CCCP (160 µM) for 24 h, respectively^42,50^. After 24-h exposure, Caspase-Glo 3–7 assay was conducted to determine levels of cleaved caspase-3, which is a direct measure of apoptosis (Supplementary Fig. 2A). As expected, there were reduced levels of cleaved caspase-3 in the DKOs compared to controls, indicating resistance to DNA (Fig. 2c) and mitochondrial toxicants (Fig. 2d). CTB assays also show the same effect with hNPCs in etoposide and CCCP exposures (Supplementary Fig. 3B, C). BAX and BAK-deficient hiPSCs and hNPCs constitute an effective model to examine the effects of disrupting developmental apoptosis during neural development.

### BAX/BAK deficiency in hiPSCs and hNPCs results in abnormal mitochondrial morphology

**Fig. 3:**
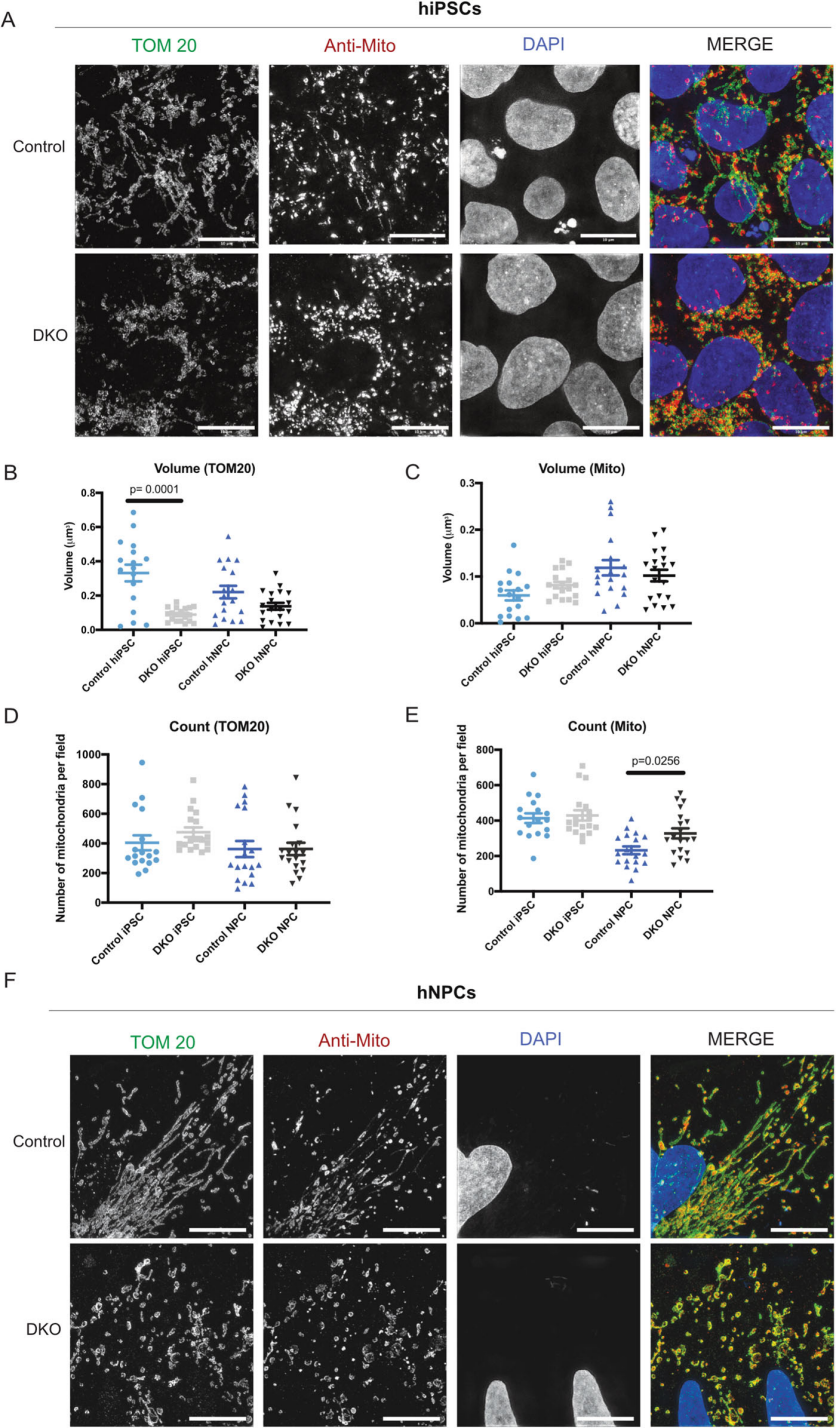
BAX/BAK DKO hiPSCs and hNPCs have abnormal mitochondrial morphology, despite having no difference in mitochondrial function. **a** TOM20 and anti-mitochondria staining in hiPSCs control and DKO shows loss of mitochondrial morphology in DKO. Scale bar = 10 µm. **b** Quantification of volume with TOM20 staining in hiPSC and hNPC lines. **c** Quantification of volume with anti-mitochondria staining in hiPSC and hNPC lines. **d** Quantification of counts using TOM20 staining in hiPSCs and hNPCs. **e** Quantification of counts using anti-mitochondria in hiPSCs and hNPCs. **f** hNPCs stained with TOM20 and anti-mitochondria show loss of mitochondrial morphology in DKO. Scale bar = 10 µm.

BAX has been previously reported to interact with proteins of the fusion and fission machinery, which are essential for mitochondrial morphology and dynamics during apoptosis^22,23,28,51^. These studies have been conducted primarily in cancer lines, but not in human stem cells. In addition, the role of BAX and BAK during homeostasis has not been explored. Using our hiPSC lines of controls and DKO, we investigated mitochondrial morphology during homeostasis using SIM. hiPSCs were fixed and stained for TOM20, an outer mitochondrial transmembrane protein, and an antibody (anti-mitochondria) generated from human cell homogenate as antigen. Our data shows that this antibody labels both the outer and inner mitochondrial membranes. Cells were imaged at ×100 using SIM (Fig. 3a). Control hiPSCs show normal, fissed mitochondria, whereas hiPSC DKOs show abnormal morphology and loss of overall mitochondrial mass. Quantification of the volume using TOM20 and anti-mitochondria antibody shows a difference between control and DKO hiPSCs (Fig. 3b, c), however, mitochondrial counts using TOM20 and anti-mitochondria antibody did not show significant difference (Fig. 3d, e). Our results suggest that loss of BAX and BAK leads to decreased mitochondrial volume when measured using TOM20. This phenomenon was previously reported in a mice study in which there was a loss of TOM20 in BAX/BAK DKO cells^52^. But loss of BAX and BAK does not seem to affect the fragmentation in hiPSCs. We then tested whether these changes in the mitochondrial network would affect mitochondrial function of the DKO hiPSCs. We used the Seahorse Mito Stress Test, which tests mitochondrial function by assessing several parameters, including basal respiration and ATP production. Three serial injections of oligomycin (inhibits ATP synthases), FCCP (disrupts proton gradient), and rotenone/antimycin A (inhibit complex I and complex III, respectively) target the electron transport chain. Control and DKO hiPSCs displayed a very similar bioenergetic profile, with no significant differences in ATP production (Supplementary Fig. 4A, B). Also, there was no difference between control and DKO hiPSCs spare respiration capacity and proton leak (Supplementary Fig. 4C, D).

We then study the effects of BAX and BAK deletion in hNPCs. We first examined the morphology of the mitochondria using SIM; control and DKO hNPCs were imaged at ×100 after fixation and staining for TOM20 and anti-mitochondria antibody (Fig. 3f). Quantification of volume with both TOM20 and anti-mitochondria show no difference between control and DKO hNPCs (Fig. 3b, c). Interestingly, there is a statistical difference with the control hNPCs having a lower count compared to DKO when stained for anti-mitochondria (Fig. 3d, e). This suggests that loss of BAX and BAK does not affect the volume of total mitochondria, but there is clearly increased mitochondrial fragmentation in the DKO cells as shown by individual counts. After measuring mitochondrial respiration and ATP levels, no significant difference was observed between the control and DKO hNPCs (Supplementary Fig. 4E, F). In addition, no differences in spare respiration capacity or proton leak were observed (Supplementary Fig. 4G, H). The experiments in hiPSCs and hNPCs support the potential role of BAX and BAK in maintaining mitochondrial morphology during homeostasis but failed to demonstrate an effect of BAX and BAK deletion on the metabolic capacity of the cells.

### BAX/BAK DKO hiPSC-derived neural rosettes show larger lumen and embryoid body size

Having observed structural differences in mitochondrial morphology in two-dimensional (2D) cellular models, we focused on determining the role of BAX and BAK in three-dimensional (3D) brain models. While BAX and BAK are well known effectors of developmental apoptosis, the results described above point to a non-canonical role of these proteins in mitochondrial morphology maintenance. Our previous report^43^ demonstrated that mutations affecting mitochondrial functionality do not impact the pluripotent capability of hiPSCs, nor the capacity of the NPCs to further differentiate into the three neural lineages in 2D model systems. However, 3D culture revealed several abnormalities consistent with the clinical phenotypes^43^.

Central nervous system (CNS) morphogenesis starts with neurulation events. This is a dynamic process by which neuroepithelial cells polarize adherens (e.g., N-cadherin) and tight junction proteins (e.g., ZO-1) toward an apical lumen while depositing extracellular matrix proteins at their basal surface^53,54^. This results in emergence of a singular neuroepithelial tube that spans the entire rostrocaudal axis of the embryo’s dorsal plane and serves as the foundation of all CNS tissues. Human pluripotent stem cells can readily differentiate into neuroepithelial cells that spontaneously polarize to form neural rosettes^55^, which are thought to recapitulate critical features of the early embryonic neural tube’s structure and function^56,57^. Further, neural rosettes can give rise to extensive levels of human corticogenesis. Thus, neural rosettes are one of the earliest in vitro models of brain development.

**Fig. 4:**
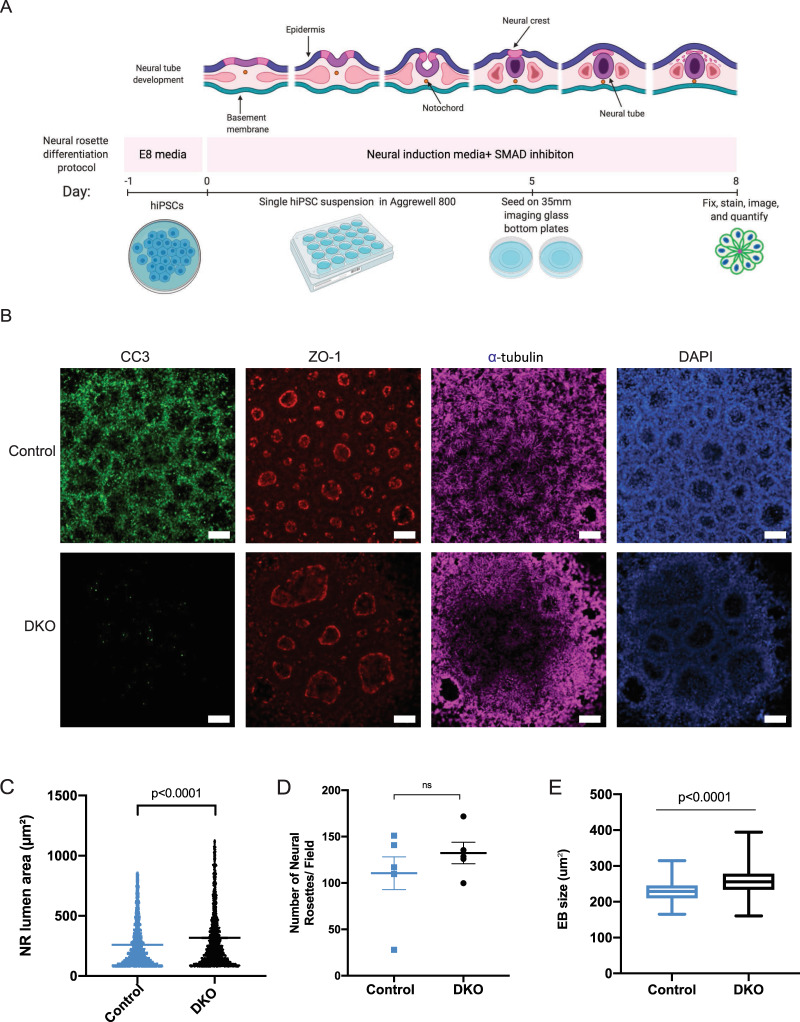
BAX/BAK DKO hiPSC-derived neural rosettes show larger lumen size and overall embryoid body size. **a** Diagram of neural rosette differentiation from hiPSCs. **b** NRs derived from EBs were stained with CC3, ZO-1, alpha-tubulin, and DAPI. DKO show virtually no CC3 compared to control. In addition, DKO present with larger and disconnected lumen. **c** Quantification of lumen size shows DKO have significantly larger lumen area compared to control. **d** No difference in NRs per field between control and DKO. **e** EB size of DKO is larger compared to control. Three independent experiments were conducted for quantification of lumen and EB size. Scale bar = 100 µm.

We obtained neural rosettes from control and DKO lines to assess the role of BAX and BAK (Fig. 4a). These structures were generated from hiPSCs, and then fixed and stained for ZO-1 (tight junction marker) and alpha-tubulin (microtubule marker) to assess overall morphology, as well as cleaved caspase-3 to measure basal levels of apoptosis (Fig. 4b). DKO neural rosettes have larger lumens (Fig. 4c) due to lack of developmental apoptosis and partial failure to close the lumen. This phenotype resembles the failure to close the neural tube observed in DKO mice^16,58^. A previous study also showed that DKO mouse models develop large numerous rosettes in SVZ^59^. The development and homeostatic defects highlighted critical roles of BAX and BAK in maintaining the functional integrity of cells and tissues. There was no difference in the number of neural rosettes per neuralized embryoid body (Fig. 4d), suggesting that increased lumen size did not affect the efficiency of neural rosette formation. In addition to increased lumen size, DKO neuralized embryoid body size was also larger potentially due to lack of cell death (Fig. 4e). These results demonstrate that deleting BAX and BAK may affect early human brain development, as reported in mouse models.

### Mature DKO cerebral organoids show loss of cortical plate markers

**Fig. 5:**
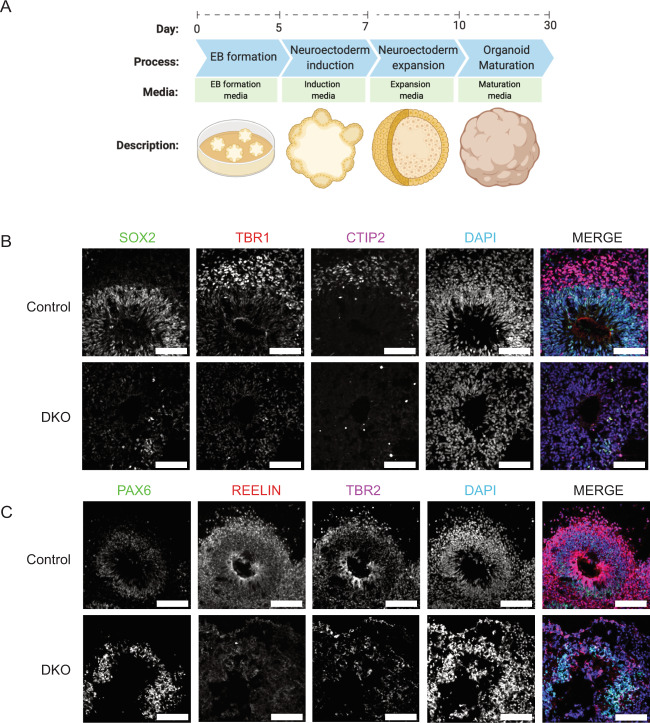
Day 30 DKO cerebral organoids show cortical disorganization and loss of cortical plate markers. **a** Differentiation protocol from hiPSCs to day 30 cerebral organoids. **b** Day 30 DKO cerebral organoids derived from hiPSCs demonstrate reduced expression of SOX2, TBR1, and CTIP2. Scale bar = 100 µm. **c** Day 30 DKO cerebral organoids have reduced expression of PAX6, Reelin, and TBR2. Scale bar = 100 µm. All day 30 experiments were conducted after three independent differentiations.

**Fig. 6:**
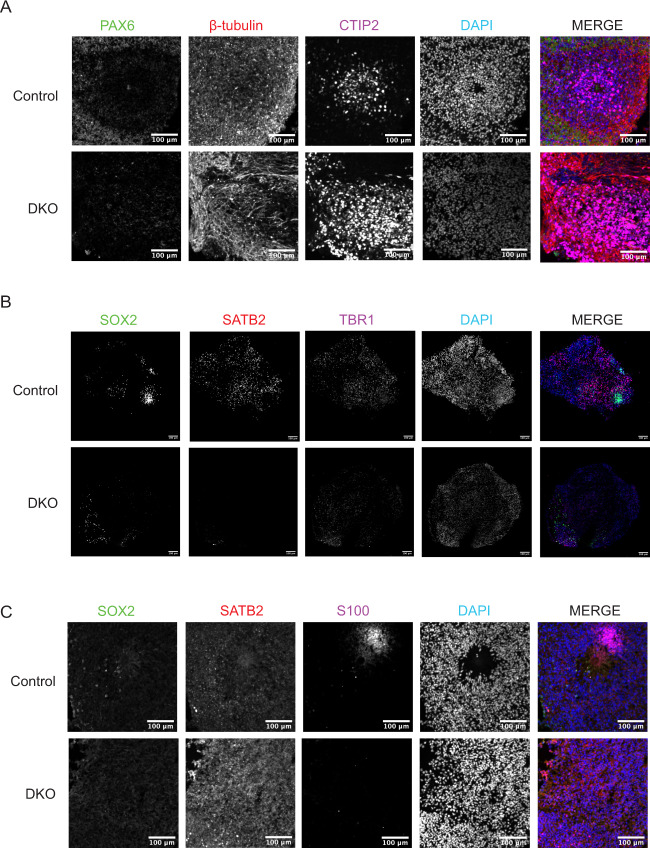
Day 60 DKO cerebral organoids have deficient neuronal marker expression. **a** Day 60 DKO cerebral organoids derived from hiPSCs demonstrate reduced expression of PAX6, β-tubulin, and CTIP2. Scale bar = 100 µm. **b** Day 60 DKO cerebral organoids have reduced expression of SOX2, SATB2, and TBR1. Scale bar = 100 µm. **c** Day 60 DKO cerebral organoids have reduced expression to SOX2, SATB2, S100. Scale bar = 100 µm. All day 60 experiments were conducted after three independent differentiations.

To determine the effects of BAX and BAK DKO in a more mature model of human development, cerebral organoids were derived from hiPSCs by neuroectoderm induction, neuroectoderm expansion, and organoid maturation under continuous agitation as previously described^60^ with some modifications^43,61^ (Fig. 5a). Cerebral organoids recapitulate many developmental processes and organization of the fetal human brain. However, as in vitro 3D brain models, they have some limitations due to lack of vascularization and neuronal maturation equivalent of the prenatal brain^62,63^. Despite these limitations, they represent an appropriate model for studying early developmental events in the brain, such as microcephaly, autism, and schizophrenia^60,64–66^. On day 30, control and DKO brain organoids were fixed, cryosectioned, and stained for phase-specific developmental markers. Transcription factors SRY-box transcription factor 2 (SOX2), Paired box protein (PAX6), and Transcription factor T-brain gene-2 (TBR2) are expressed in neuroepithelial and radial glia cells in the ventricular zone. To identify deep layer neurons, organoids were stained with T-Box brain transcription factor 1 (TBR1) and Chicken ovalbumin upstream promoter transcription factor-interacting protein (CTIP2), which mark cortical plate layer six (VI) and five (V). Layer one (I) was marked using REELIN, which is expressed in the transient Cajal–Retzius cells and present in the marginal zone. Controls show proper development and organization of neural progenitors and layers V and VI neurons. However, DKOs display a profound disorganization of cortex, as well as a marked loss of TBR1 and CTIP2 (Fig. 5b, c). Several reports show increase in neuronal stem cell population in DKO mice^13,67,68^. Thus, to further study the disrupted cortical development in the DKOs, we derived day 60 cerebral organoids. These were stained and imaged for neural progenitor markers (PAX6, SOX2) as well as neuronal markers (β-tubulin, CTIP2, Special AT-rich sequence-binding protein 2 (SATB2), TBR1, and S100 calcium-binding protein (S100)) (Fig. 6a–c). Similar to day 30, day 60 organoids showed decreased expression of PAX6 in DKOs and SOX2, as early development is concluding. Neuronal markers CTIP2, SATB2, TBR1, and S100 all show decreased expression in DKO. No difference was observed between levels of a general neuronal marker β-tubulin.

### Mature DKO cerebral organoids have abnormal mitochondrial morphology

**Fig. 7:**
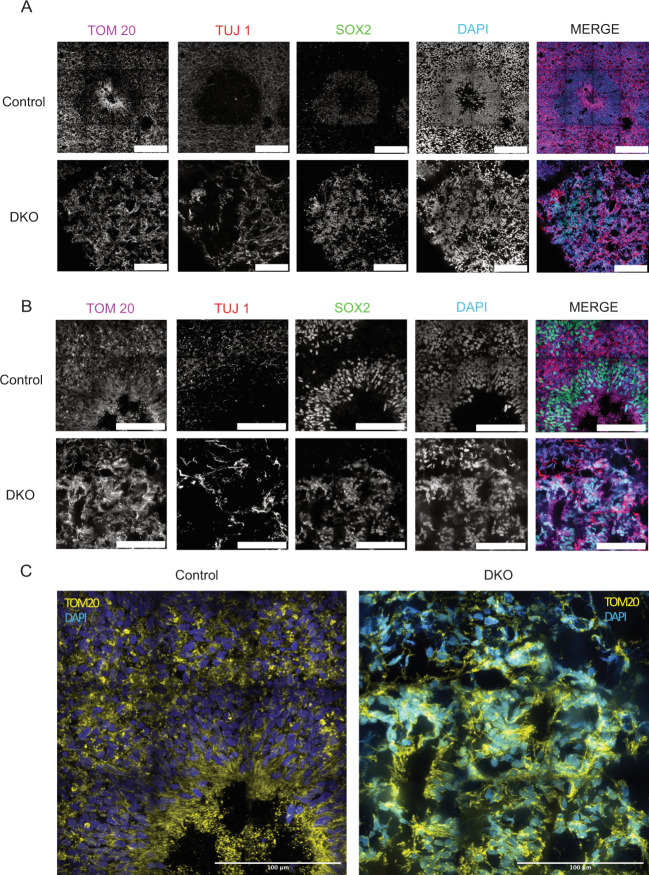
DKO cerebral organoids have abnormal mitochondrial morphology. **a** DKO cerebral day 30 organoids stained with TOM20, TUJ1, and SOX2 show disorganized mitochondrial morphology in a 4 × 4 stitched image. Scale bar = 100 µm. **b** Higher magnification of cerebral day 30 organoids shows abnormal and aggregated mitochondria in DKO in a 3 × 3 stitched image. **c** Zoomed insert of control and DKO shows the intricate mitochondrial networks and the differences that are present. All day 30 experiments were conducted after three independent differentiations.

**Fig. 8:**
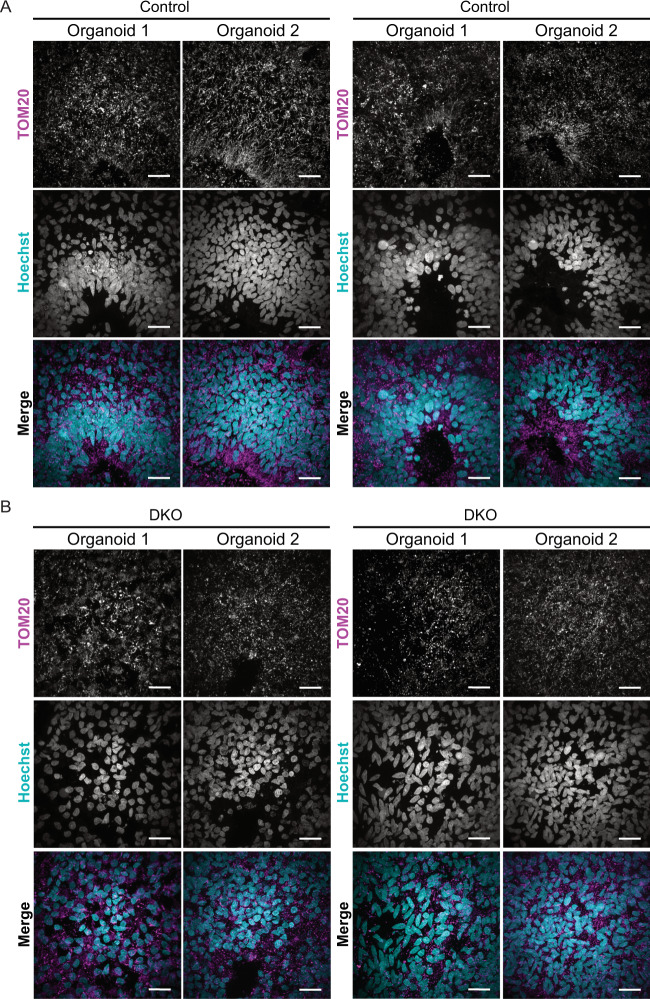
Day 60 DKO cerebral organoids have abnormal mitochondrial morphology. **a** Control organoids stained with TOM20 show fragmented and elongated mitochondria. Scale bar = 100 µm. **b** DKO organoids stained with TOM20 show fragmented and disrupted mitochondria. Scale bar = 100 µm.

Day 30 organoids were stained for TOM20 to examine mitochondrial morphology. Control organoids show elongated mitochondrial morphology in the ventricular zone and fragmented mitochondrial morphology in newly committed neurons as shown in murine models^69^, suggesting that in both humans and mice, the mitochondrial network morphology is differentially regulated between the progenitor zones and the cortical plate. Previous studies have shown that commitment of NPCs to a neurogenic fate is accompanied by morphological and metabolic changes of the mitochondria^29,70^. BAX and BAK DKO brain organoids show striking aggregation of mitochondrial networks and abnormal morphology at this stage (Fig. 7a, b). Zoomed inserts of control and DKO organoids with TOM20 staining show the differences in mitochondrial morphology (Fig. 7c). Day 60 brain organoids were also stained and imaged for TOM20 to determine differences in mitochondrial morphology in a later developmental stage. Across controls, individual organoids show fragmented mitochondria in the progenitor zone and elongated mitochondria in layers of the cortex (Fig. 8a, b). The DKO brain organoids, on the other hand, show more fragmented, disrupted mitochondria, and reduced lumen areas corresponding to the neural progenitor zone (Fig. 8c, d). The results from these brain models highlight the importance of BAX and BAK to maintain mitochondrial morphology during early brain development.

## Discussion

BAX and BAK are well known executioners of developmental apoptosis with overlapping roles. They also appear to mediate mitochondrial dynamics during cell death. Previous studies have used mouse models and immortalized cell lines to study BAX and BAK functions^9,13,16,21–23,51^. These studies increased our understanding of how BAX and BAK mechanistically regulate mitochondrial-mediated apoptosis. However, there is a lack of data in human model systems.

Here, we studied the effects of proapoptotic proteins BAX and BAK in mitochondrial morphology during homeostasis in human iPSC model systems. First, hiPSCs were genetically modified using CRISPR-mediated gene editing to generate BAX/BAK DKO iPSCs and hNPCs. We confirmed these cells were resistant to cell death by exposure to DNA-damaging agent etoposide and mitochondrial toxicity agent CCCP. Apoptosis/necrosis assays with show a decrease in apoptosis in DKO iPSCs. BOK, which has been shown to carry out apoptosis in the absence of BAX and BAK^20^, may be responsible for the low levels of apoptosis detected in these lines. BOK has been shown to be unresponsive to antiapoptotic proteins of the BCL-2 family^17^. A recent study implicates BOK in maintenance of mitochondrial morphology and shows that BOK deletion or depletion significantly reduces mitochondrial fusion, resulting in fragmented mitochondria^71^. However, more studies with BOK are needed to appreciate its contribution to apoptosis and potential roles in modulating mitochondrial morphology.

After validating that DKO hiPSC and differentiated hNPC lines are resistant to apoptosis, mitochondrial morphology was assessed using SIM confocal imaging to examine the effects of BAX and BAK deletion on mitochondrial morphology. DKO hiPSCs and hNPCs show dramatic mitochondrial network dysfunction suggesting that BAX and BAK deletion affects the maintenance of mitochondrial morphology. These proapoptotic proteins have been previously implicated in mediating mitochondrial function and structure^21,22,24,25,31,72^, and our results support a similar role for BAX and BAK in modulating mitochondrial morphology in these hiPSC-derived systems. Unexpectedly, there was not a statistical difference in the levels of oxidative phosphorylation or ATP levels between control and DKO cells, which indicates that despite abnormal mitochondrial morphology, these DKO cells are still capable of ATP production. For mitochondrial respiration to occur efficiently, a cell needs sufficient inner mitochondrial membrane surface area, suggesting that DKO cells may have an intact inner mitochondrial membrane, but defects in outer mitochondrial membrane. Interestingly, previous study in mouse embryonic fibroblasts (MEFs) shows that respiration in BAX/BAK DKO does not change compared to WT MEFs^73^. Additional assessment of mitochondrial inner membrane morphology, cristae dynamics, and mitochondrial biogenesis could provide additional insight into the effects of BAX/BAK deletion on mitochondrial homeostasis.

While extremely useful, 2D cultures are unable to fully represent complex brain tissue organization or regional identity^74^. DKO neural rosettes, which mimic the neural tube formation in vivo, have larger lumens and irregular morphology. Midline fusion in vivo has been shown to be dependent on developmental apoptosis^58^. Loss of BAX and BAK in mouse models is embryonic lethal partially due to failure to close the neural tube^16,58^. Neural tube closure consists of complex cellular events such as convergent extension, apical constriction, and interkinetic nuclear migration, in addition to activation of Wnt/planar cell polarity and Shh/BMP cells signaling pathways^75^. If developmental apoptosis does not occur, neural tube defects arise, often leading to severe disabilities similar to those seen in spina bifida. Our results in 3D human models suggest that deletion of BAX/BAK results in failure to close neural rosettes, resembling the phenotype described in DKO and TKO mouse models. Our unpublished data show that inhibition of caspases using qVD-Oph did not affect differentiation capacity in hiPSCs as previously shown for mouse embryonic stem cells^76^. These results suggest that the neural rosette deficiencies may be due to the loss of the mitochondrial network rather than to the loss of apoptosis. Future studies should examine caspase deficient models to confirm this possibility.

Corticogenesis, the process by which the cerebral cortex is formed into six layers, requires a series of complex and well-coordinated developmental steps. Abnormalities in cortical development can lead to a complex set of malformations observed in cerebral development disorders, like severe intellectual disability and autism^77^. BAX/BAK DKO cerebral organoids had fewer number of deep layer neurons specifically in layers V and VI. Neural progenitor makers (PAX6 and SOX2), cortical plate markers (TBR1 and CTIP2), radial glia marker (TBR2), and layer I/marginal zone marker (REELIN) were used to characterize development at day 30. These markers allow for cell labeling and help determine location and organization within the cortex. Our data show that loss of BAX and BAK causes severe perturbations in corticogenesis, leading to loss of distinct organization and improper cell development. Interestingly, cells in DKO organoids have decreased expression of neural progenitor and neuronal markers found during normal corticogenesis. This suggests that lack of BAX and BAK does not induce these cells to undergo apoptosis but rather to continue maturing and possibly limit the growth of necessary cell types. These are perhaps cells types of other lineages which may have diverged due to lack of BAX and BAK. Excessive fission has been associated with mtDNA loss and increase ROS levels^78–82^, which cannot be excluded as triggers of the corticogenesis defects detected in DKO brain organoids. Additional studies should be performed to further explore the molecular mechanism underlying the abnormal corticogenesis in DKO. Overall, our data support an essential function for BAX and BAK in normal neurodevelopment. The drastic differences in mitochondrial morphology at days 30 and 60 in DKO cerebral organoids suggests that BAX and BAK may be critical modulators of mitochondrial morphology and that disruption of the mitochondrial network is a critical driver of this phenotype.

A study in murine models showed that mitochondrial dynamics regulates neurogenesis^83^. Cells with high levels of mitochondrial fission were more likely to become neurons whereas cells with increased mitochondrial fusion undergo self-renewal. This study reported that postmitotic control of cell fates via mitochondrial dynamics is conserved in both mouse and human corticogenesis, assessed through the analysis of human embryonic stem cell-derived cortical neurons^83^. This report demonstrates that neural progenitors of the ventricular zone maintain an elongated mitochondrial morphology that progressively fragments as differentiation advances. These results are significant and indicate that iPSC-derived brain organoids support the developmental remodeling of the mitochondria described in vivo^29^. Future studies will aim to understand the cell fate and metabolic consequences of BAX and BAK deletion in the context of early brain development and mitochondrial remodeling using human brain organoids BAX and BAK have been shown to modulate mitochondrial proteins involved in fusion^25,84^ and fission^21–23,28^. Studies in animal models revealed the importance of mitochondrial dynamics during neurogenesis and stem cell fate decisions^29,69,70^. Therefore, we speculate that BAX and BAK can also modulate cell fate and differentiation through mechanisms that remain to be elucidated.

Our findings indicate that besides the role of BAX and BAK in regulating developmental apoptosis, these proteins may also support mammalian brain development by maintaining the morphology of the mitochondrial network. Future studies should aim to determine the exact mechanisms by which BAX and BAK deletion leads to abnormal mitochondrial morphology in hiPSCs and hNPCs during homeostatic development. In addition to BAX and BAK, BOK, which is structurally homologous to other proapoptotic proteins, seems to play similar roles in mitochondrial morphology^71^. Characterizing the function of these BCL-2 effectors will improve our understanding of mitochondrial dysfunction in human development and disease.

## Supplementary information

Supplemental Figure 1

Supplemental Figure 2

Supplemental Figure 3

Supplemental Figure 4

Supplemental Figure Legends
