# Supplementary figures and images for: Modeling the function of BAX and BAK in early human brain development using iPSC-derived systems
Source: Cell Death Dis. 2020 Sep 25;11(9):808. doi: 10.1038/s41419-020-03002-x (PMC7519160; doi:10.1038/s41419-020-03002-x)

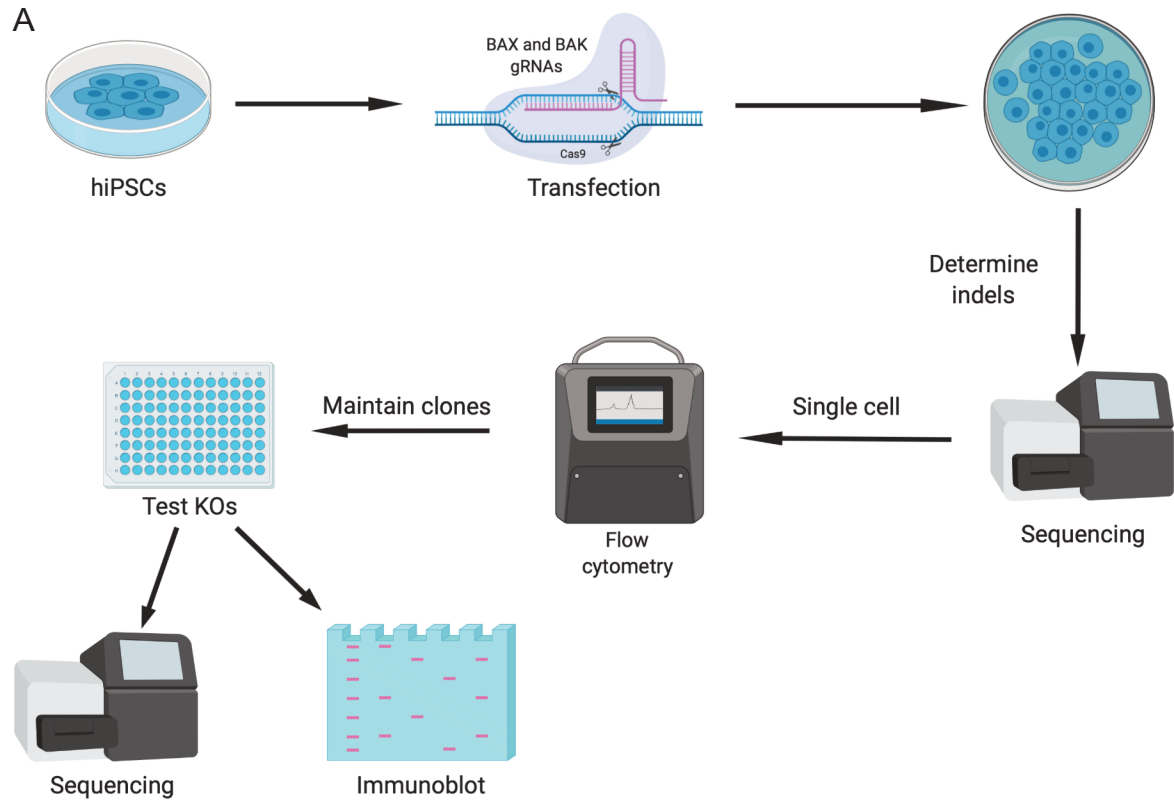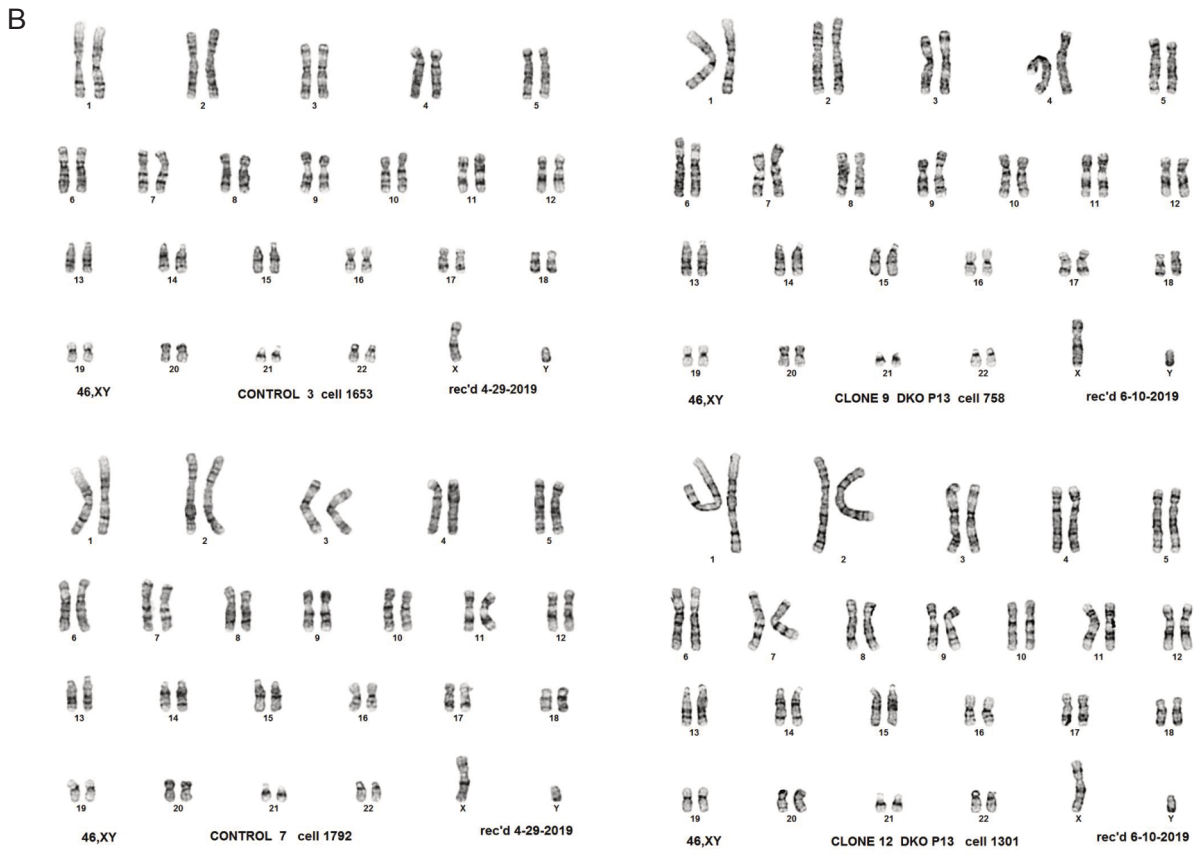

Supplement: Supplementary file 1 — Supplemental Figure 1 [file 41419_2020_3002_MOESM1_ESM.pdf]

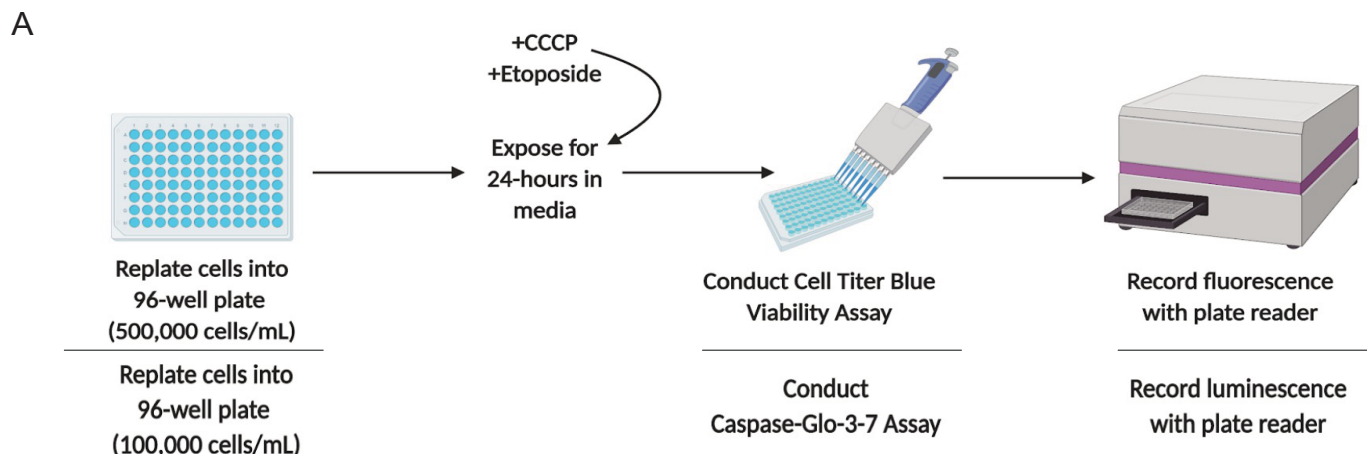

**B** CC3 Assay: Etoposide exposure in hiPSCs- 24 hour

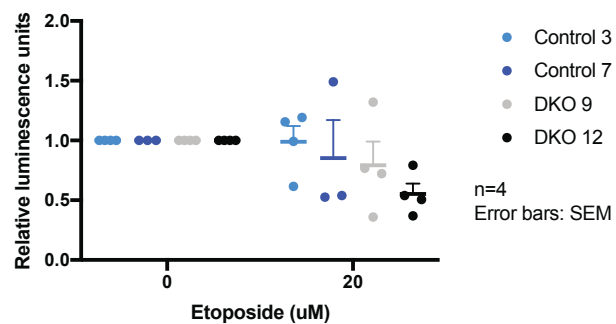

**C** CC3 Assay: CCCP exposure in hiPSCs -24 hours

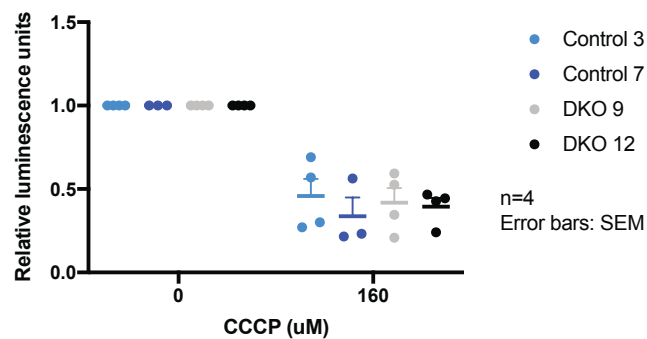

Supplement: Supplementary file 2 — Supplemental Figure 2 [file 41419_2020_3002_MOESM2_ESM.pdf]

Supplemental Figure 4

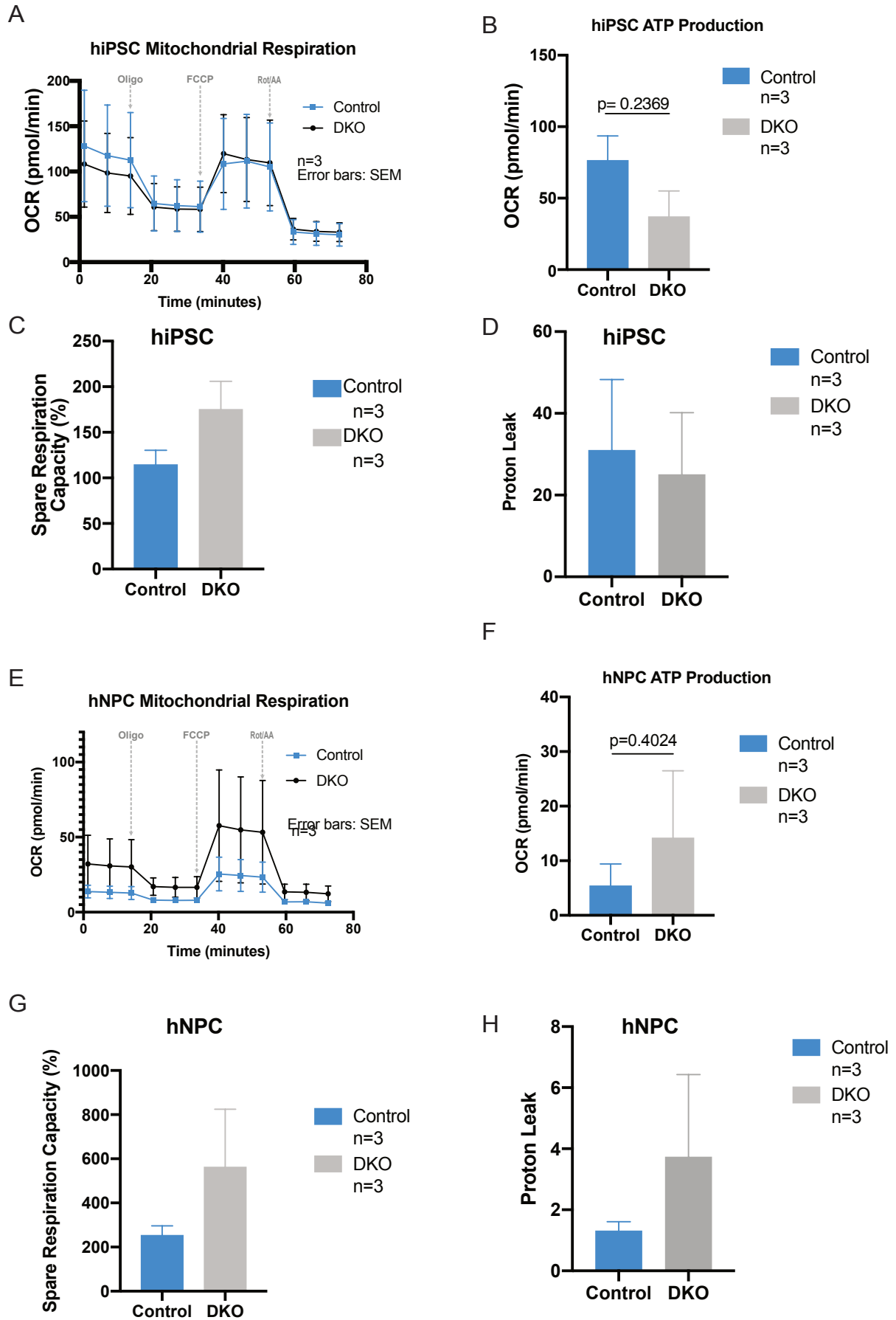

Supplement: Supplementary file 4 — Supplemental Figure 4 [file 41419_2020_3002_MOESM4_ESM.pdf]
