# Supplementary material for: Modeling the function of BAX and BAK in early human brain development using iPSC-derived systems
Source: Cell Death Dis. 2020 Sep 25;11(9):808. doi: 10.1038/s41419-020-03002-x (PMC7519160; doi:10.1038/s41419-020-03002-x)
Supplement: Supplementary file 3 — Supplemental Figure 3 [file 41419_2020_3002_MOESM3_ESM.pdf]

A

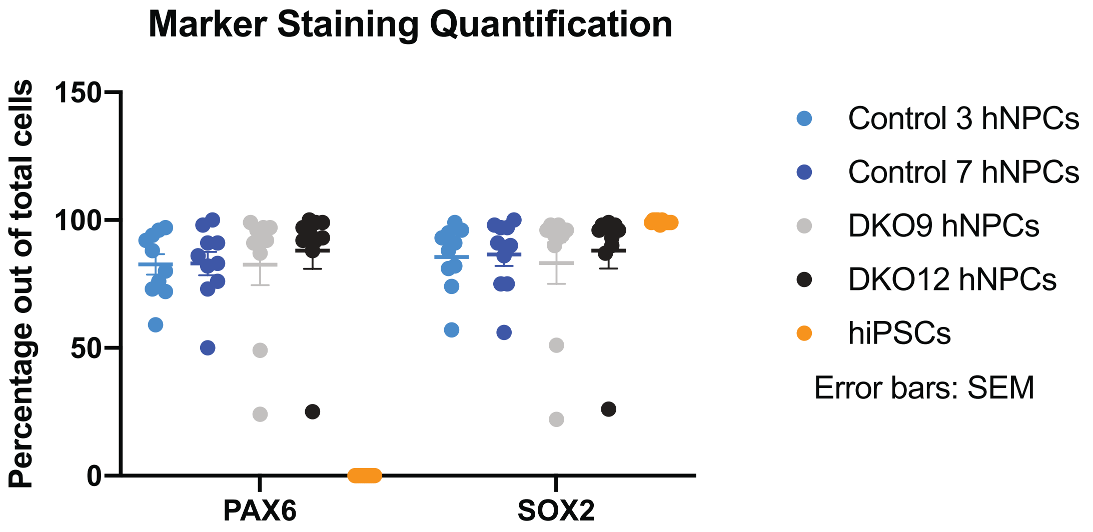
Marker Staining Quantification
Percentage out of total cells
150
100
50
0
PAX6
SOX2
Control 3 hNPCs
Control 7 hNPCs
DKO9 hNPCs
DKO12 hNPCs
hiPSCs
Error bars: SEM

B

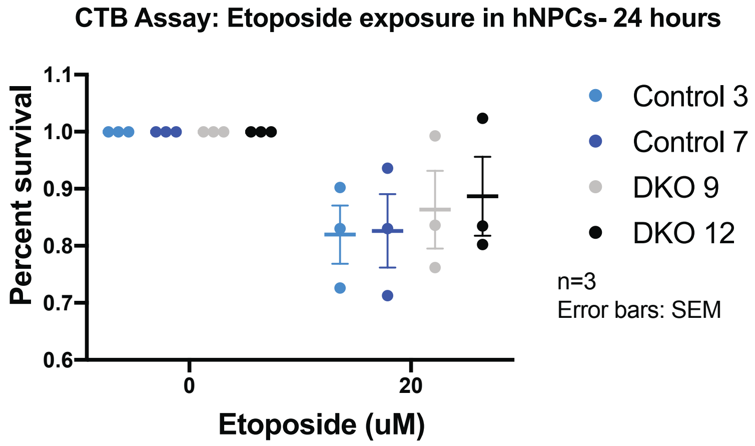
CTB Assay: Etoposide exposure in hNPCs- 24 hours
Percent survival
1.1
1.0
0.9
0.8
0.7
0.6
0
20
Etoposide (uM)
Control 3
Control 7
DKO 9
DKO 12
n=3
Error bars: SEM

C

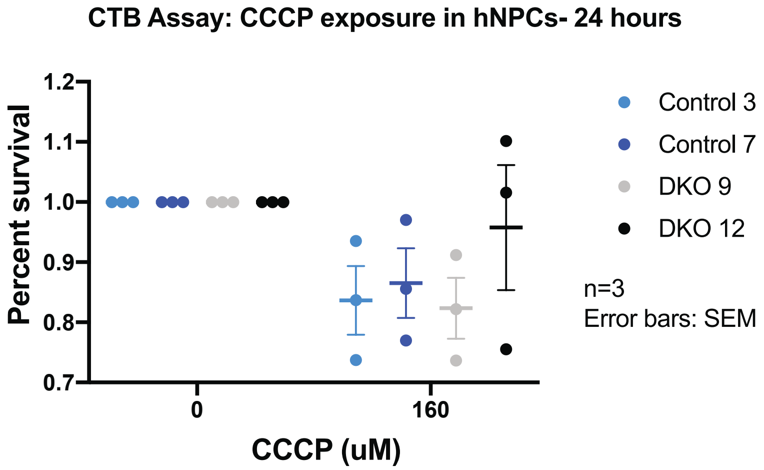
CTB Assay: CCCP exposure in hNPCs- 24 hours
Percent survival
1.2
1.1
1.0
0.9
0.8
0.7
0
160
CCCP (uM)
Control 3
Control 7
DKO 9
DKO 12
n=3
Error bars: SEM
