# Supplementary material for: Modeling the function of BAX and BAK in early human brain development using iPSC-derived systems
Source: Cell Death Dis. 2020 Sep 25;11(9):808. doi: 10.1038/s41419-020-03002-x (PMC7519160; doi:10.1038/s41419-020-03002-x)
Supplement: Supplementary file 5 — Supplemental Figure Legends [file 41419_2020_3002_MOESM5_ESM.pdf]

Supplemental Figure Legends

Supplemental Figure 1: CRISPR protocol for hiPSCs and normal karyotype for edited hiPSC lines. (A) CRISPR protocol that was used for generating DKO clones. (B) Two control and two DKO lines utilized in this study show normal karyotype.

Supplemental Figure 2: Exposure paradigm and hiPSCs toxicant exposures measured with cleaved caspase 3 assay. **(**A) Exposure paradigm for using cell titer blue (CTB) and cleaved caspase 3 (CC3) assay with hiPSCs and hNPCs. (B) Cleaved caspase 3 assay in hiPSCs with etoposide. (C) Cleaved caspase 3 assay in hiPSCs with CCCP. Four independent experiments were conducted for etoposide and CCCP exposures. Each symbol represents an individual experiment and the percent survival for that clone. Error bars: SEM.

Supplemental Figure 3: Validation of neurodifferentiation and hNPCs toxicant exposures measured with cell titer blue. (A) hNPC and hiPSC PAX6 and SOX2 quantification. (B) Cell titer blue assay in hNPCs with etoposide. (E) Cell titer blue assay in hNPCs with CCCP. Three independent experiments were conducted for etoposide and CCCP exposures. Each symbol represents an individual experiment and the percent survival for that clone. Error bars: SEM.

Supplemental Figure 3: Seahorse experimental data for respiration, ATP production, Spare respiration capacity, and proton leak for hiPSCs and hNPCs. (A) Seahorse mito stress test also suggests no difference in mitochondrial respiration after three independent experiments in hiPSCs. (B) Seahorse assay results show there is no difference in ATP production between control and DKO after three independent experiments in hiPSCs. (C) Spare respiration capacity (%) show no difference in hiPSCs. (D) hiPSCs proton leak shows no difference between control and DKO. (E) Seahorse mito stress test also shows no difference in OCR after 3 independent experiments in hNPCs. (F) Seahorse assay results demonstrate no difference in ATP production in hNPCs. (G) hNPCs were measured for spare respiration capacity and show no difference. (H) hNPCs proton leak shows no difference between DKO and control.
